# Virgin Olive Oil and Health: Summary of the III International Conference on Virgin Olive Oil and Health Consensus Report, JAEN (Spain) 2018

**DOI:** 10.3390/nu11092039

**Published:** 2019-09-01

**Authors:** José J. Gaforio, Francesco Visioli, Catalina Alarcón-de-la-Lastra, Olga Castañer, Miguel Delgado-Rodríguez, Monserrat Fitó, Antonio F. Hernández, Jesús R. Huertas, Miguel A. Martínez-González, Javier A. Menendez, Jesús de la Osada, Angeliki Papadaki, Tesifón Parrón, Jorge E. Pereira, María A. Rosillo, Cristina Sánchez-Quesada, Lukas Schwingshackl, Estefanía Toledo, Aristidis M. Tsatsakis

**Affiliations:** 1 Center for Advanced Studies in Olive Grove and Olive Oils, University of Jaen, 23071 Jaén, Spain; 2 Department of Health Sciences, Faculty of Experimental Sciences, University of Jaén, 23071 Jaén, Spain; 3 Agri-Food Campus of International Excellence (ceiA3), 14071 Córdoba, Spain; 4 CIBER Epidemiología y Salud Pública (CIBER-ESP), Instituto de Salud Carlos III, 28029 Madrid, Spain; 5 Department of Molecular Medicine, University of Padova, 35121 Padova, Italy; 6 Laboratory of Functional Foods, Instituto Madrileño de Estudios Avanzados (IMDEA)-Alimentación, CEI UAM + CSIC, 28049 Madrid, Spain; 7 Department of Pharmacology, Faculty of Pharmacy, University of Seville, 41012 Sevilla, Spain; 8 Cardiovascular Risk and Nutrition Research Group (CARIN), Hospital del Mar Medical Research Institute (IMIM), 08003 Barcelona, Spain; 9 CIBER Obesity and Nutrition (CIBER-OBN), Instituto de Salud Carlos III, 28029 Madrid, Spain; 10 Department of Legal Medicine and Toxicology, University of Granada School of Medicine, 18016 Granada, Spain; 11 Institute of Nutrition and Food Technology, Biomedical Research Centre, Department of Physiology, Faculty of Sport Sciences, University of Granada, 18071 Granada, Spain; 12 Department of Preventive Medicine and Public Health-IdiSNA, University of Navarra, 31008 Pamplona, Spain; 13 Department of Nutrition, Harvard TH Chan School of Public Health, Boston, MA 02115, USA; 14 ProCURE (Program Against Cancer Therapeutic Resistance), Metabolism and Cancer Group, Catalan Institute of Oncology, 17007 Girona, Spain; 15 Girona Biomedical Research Institute (IDIBGI), 17190 Girona, Spain; 16 Department of Biochemistry, Molecular and Cellular Biology, Veterinary Faculty, University of Zaragoza, 50013 Zaragoza, Spain; 17 Centre for Exercise, Nutrition and Health Sciences, School for Policy Studies, University of Bristol, Bristol BS8 1TZ, UK; 18 Departamento de Enfermería, Fisioterapia y Medicina, Universidad de Almería, 04120 Almería, Spain; 19 Facultad de Agronomía, Universidad de la República, 12900 Montevideo, Uruguay; 20 Institute for Evidence in Medicine, Medical Center – University of Freiburg, Faculty of Medicine, University of Freiburg, 79110 Freiburg, Germany; 21 Laboratory of Toxicology, Medical School, University of Crete, 71003 Heraklion, Crete, Greece

**Keywords:** olive oil, polyphenols, hydroxytyrosol, Mediterranean diet, cardiovascular disease, cancer, neurodegeneration, sustainable diet, sustainable agriculture

## Abstract

The Mediterranean diet is considered as the foremost dietary regimen and its adoption is associated with the prevention of degenerative diseases and an extended longevity. The preeminent features of the Mediterranean diet have been agreed upon and the consumption of olive oil stands out as the most peculiar one. Indeed, the use of olive oil as the nearly exclusive dietary fat is what mostly characterizes the Mediterranean area. Plenty of epidemiological studies have correlated that the consumption of olive oil was associated with better overall health. Indeed, extra virgin olive oil contains (poly)phenolic compounds that are being actively investigated for their purported biological and pharma-nutritional properties. On 18 and 19 May 2018, several experts convened in Jaen (Spain) to discuss the most recent research on the benefits of olive oil and its components. We reported a summary of that meeting (reviewing several topics related to olive oil, not limited to health) and concluded that substantial evidence is accruing to support the widespread opinion that extra virgin olive oil should, indeed, be the fat of choice when it comes to human health and sustainable agronomy.

## 1. Introduction

The Mediterranean dietary pattern is as an excellent model of healthy eating. The term “Mediterranean diet” (MedDiet) was coined by Ancel Keys in the early 1960s and is based on the traditional culinary practices of rural areas of Southern Italy (where he lived), Crete, other parts of Greece, and other countries of the Mediterranean basin in the 1950s–1960s [1].

There are some geographical differences in the traditional Mediterranean dietary pattern, but they all share the use of olive oil as the main culinary fat (1). Other elements included in the most frequently used definition of the MedDiet include high consumption of fruits and nuts (2), vegetables (3), legumes (4), fish (5), and whole grain cereals (6). Furthermore, a salient element of this definition is low consumption of red meat and processed meats (7) and moderate or low consumption of dairy products (8). Alcohol is consumed in modest amounts, mostly as red wine with meals (9). These nine features are used to operationally define the MedDiet as proposed by Trichopoulou et al. [2], who used the sex-specific medians of the first six elements to assign 1 point for each element when it is above the sex-specific median and the other two elements (meat and dairy) are used to assign 1 point if they are below the sex-specific median; an additional point is assigned if ethanol intake is in the range of 5–25 g/d for women and 10–50 g/d for men. Other authors have used tertiles instead of dichotomizing by medians.

Many meta-analyses have summarized the findings of large and well-conducted cohort studies showing the benefits of a better adherence to a MedDiet for the prevention of non-communicable disease (NCD) [3,4,5,6,7]. Among the different cohorts that have assessed associations between adherence to the MedDiet and NCD, the Seguimiento Universidad de Navarra (SUN) project is a large and still ongoing prospective cohort of relatively young adults at baseline that was designed to assess the effects of the MedDiet in a Mediterranean setting [8]. Considering both a high adherence to the MedDiet and a physically active lifestyle, the SUN is reporting a very strong reduction in all-cause mortality [9].

The benefits of adhering to a MedDiet for the prevention of NCD have also been proven by two controlled clinical trials. One, the Lyon Diet Heart trial, used a MedDiet enriched with alpha-linolenic acid in survivors of a myocardial infarction and showed a dramatic reduction in reinfarctions compared to a control group [10]. The PREDIMED trial, an intervention with a MedDiet in primary cardiovascular prevention with either extra-virgin olive oil or mixed nuts, showed a 30% relative risk reduction in the occurrence of a first clinical cardiovascular event compared to a control group, where participants were recommended to follow a low-fat diet [11,12].

As mentioned above, olive oil, particularly extra-virgin olive oil, is the most characteristic component of the MedDiet. Several experts convened in Jaen (Spain) on 18 and 19 May 2018 to discuss the most recent research on olive oil and its components. What follows is a summary of that meeting.

## 2. Future Challenges to Public Health and the Role of Virgin Olive Oil

The Global Burden of Disease analyzes the relative contribution of different causes of mortality [13]. In the Western world, NCDs account for the majority of deaths and cardiovascular diseases (CVD) are still the number one contributor. Diet and lifestyle play a major preventive role.

In terms of risk factors, the three world leading factors for CVD are (a) high systolic blood pressure (SBP), (b) smoking, and (c) high body mass index (BMI). For men, high SBP is the leading risk factor in nearly all high-income countries [13,14]. On average, global population SBP decreased slightly since 1980, but trends varied significantly across regions and countries, with significant decreases in North America, Western Europe and Australasia. SBP is currently highest in low-income and middle-income countries [15].

Hypertension independently increases the risk of coronary heart disease, sudden death, stroke, heart failure, and peripheral arterial disease. Several epidemiological studies have analyzed the relationship between monounsaturated fatty acids (MUFA) consumption and hypertension [16]. The OmniHeart study—published in 2005—compared three diets: one rich in carbohydrates, another one rich in vegetable proteins, and a third diet rich in MUFA. The diets rich in vegetable proteins and MUFA-compared with the carbohydrate one-reduced blood pressure and improved lipid profiles [17]. The International Study of Macro/Micronutrients and Blood Pressure (INTERMAP) is a multi-centre cross-sectional study of 4680 men and women that reported a significant inverse relationship between total MUFA intake and diastolic blood pressure (DBP) [18]. A Spanish study showed that intakes of 13 g/d of oleic acid from vegetables was associated with reductions in both SBP and DBP [18]. In the PREDIMED, when assessing 24-h ambulatory blood pressure, SBP decreased −2.3 mm Hg (95% confidence interval (CI), −4.0–−0.5) and DBP decreased −1.2 mm Hg (95% CI, −2.2–−0.2) with extra-virgin olive oil after one year of follow-up [19].

A systematic review was recently published that evaluated clinical trials of olive oil and its effects on blood pressure in individuals that were free of cardiovascular events. The paper reported that olive oil diets led to a decrease in blood pressure, while capsules providing different amounts of EVOO did not produce any effect. In particular, DBP decreased, on average, by −0.73 mm Hg (95% CI, −1.07–−0.40)) after the use of olive oil. This reduction was mainly due to extra virgin olive oil (EVOO) consumption from 10 to 50 mL per day: −1.44 mm Hg (95% CI, −1.89–−1.00); *p* < 0.001. However, the decrease was not statistically significant for SBP [20].

Obesity is the current most important pandemic. High BMI is the leading risk factor in women in nearly all countries in the Americas, north Africa, and the Middle East, and in many other high-income countries [21]. In Western Europe, BMI recently increased by 20% in males and 18% in females [22]. High BMI is an important risk factor for mortality and morbidity from CVD, diabetes, cancers, and musculoskeletal disorders [23,24].

Several prospective studies e.g., the EPIC [25,26] and the SUN analyzed whether the MedDiet was related to weight and BMI [27]. They were pooled in several meta-analyses [28,29] that associated adherence to MedDiet with a long-term reduction in BMI. This association was stronger when energy restriction was also applied. In addition, the PREDIMED trial, which allowed ad libitum total energy intake, showed a slight reduction in body weight gain after a median 5-year follow-up [30]. Several observational studies have also associated olive oil use with lower BMI, independently of the diet [31,32]. As per a systematic review, a diet enriched with olive oil reduced weight more than control diets: −0.92 kg (95% CI, −1.16–−0.67) and diminished BMI by −0.90, (95% CI, −0.91–−0.88), *p* heterogeneity < 0.001. The benefits were seen when olive oil was supplemented as part of the overall dietary pattern and not when given via capsules [33].

## 3. Extra-Virgin Olive Oils and Prevention of NCDs

### 3.1. Extra-Virgin Olive Oil and Cancer

#### 3.1.1. Breast Cancer

Breast cancer is the second most frequently diagnosed malignant tumor and it is the most common malignancy among women [34]. Worldwide, more than 2 million new breast cancer diagnoses were made in 2018. In terms of mortality, breast cancer is the fifth cause of death among different tumor types (627,000 deaths estimated in the world in 2018) and the third cause of death among women in developing countries, and the fourth one in the most developed areas [35].

Historically, a lower incidence of breast cancer has been observed in Mediterranean countries than in other European countries or the USA [36]. In this sense, diet might play a role in breast cancer incidence because the diets of these countries are quite different.

In a meta-analysis of the association between a higher adherence to the traditional MedDiet and the incidence of postmenopausal breast cancer, including data from cohort studies, the combined estimate for overall postmenopausal breast cancer incidence was 0.94 (95% CI 0.88–1.01) [37]. A significant inverse association was observed when a higher vs. a lower adherence to the traditional MedDiet were compared for estrogen receptor negative (ER-) breast cancer (Hazard Ratio (HR) 0.60 (95% CI, 0.39–0.93)). Even though moderate alcohol use (preferably in the form of red wine and consumed with meals) is a characteristic trait of the MedDiet, there is strong evidence suggesting that higher alcohol consumption is associated with a higher risk of breast cancer [38]. In the aforementioned meta-analysis, two out of the five studies incorporated alcohol consumption in the assessment of the adherence to the traditional MedDiet. Interestingly, when the studies in which the alcohol intake component for calculating the adherence to the MedDiet had not been considered, the HR for postmenopausal breast cancer incidence among women with a higher adherence to the traditional MedDiet compared to women with a lower adherence became 0.92 (95% CI 0.87–0.98). It is also noteworthy that only one of the studies included in the meta-analysis included participants from Mediterranean countries. This latter study was done in the European Prospective Investigation into Cancer and Nutrition (EPIC) cohort [39] by Buckland and colleagues and included participants from Italy, Greece and Spain. The authors found that better adherence to the traditional MedDiet was associated with a significant 7% reduction in the risk of postmenopausal breast cancer, compared to women in the lowest adherence group (HR = 0.93, 95% CI 0.87–0.99).

With regards olive oil, to our knowledge, there is only one prospective cohort study that assessed the association between olive oil consumption and the risk of breast cancer [40]. In that study, data from Mediterranean countries from the EPIC cohort were included and no significant association was observed. Nevertheless, it has to be underscored that no distinction between varieties of olive oil was done, i.e., no specific association between extra-virgin olive oil consumption or refined olive oils consumption and the risk of postmenopausal breast cancer was reported. In addition, evidence from case-control studies suggests that olive oil intake could be inversely associated with the risk of breast cancer [41].

In the PREDIMED trial, participants at high CVD were allocated in a 1:1:1 ratio to a nutritional intervention fostering the adherence to a traditional MedDiet supplemented with either extra-virgin olive oil or mixed nuts or to a control group. Participants in the control group were advised to follow a low-fat diet [42]. The main outcome in the trial was the incidence of CVD, but incidence of breast cancer was included as a secondary outcome [43]. Among 4152 women included in the analysis, women allocated to the MedDiet supplemented with extra-virgin olive oil group exhibited a 68% relatively lower risk of incident breast cancer (HR = 0.32, 95% CI 0.13–0.79) compared to the control group. Women allocated to the MedDiet group supplemented with nuts showed a HR = 0.59 (95% 0.26–1.35), compared to the control group. In particular, dose-response observational analyses found a HR of 0.72 (95% CI 0.57–0.90) for each additional 5% of calories from extra-virgin olive oil. In terms of breast cancer, the main limitation of the PREDIMED trial was the small number of observed incident cases (only 35). However, the observed risk reduction with extra-virgin olive oil was so dramatic that even with such small number of cases, differences were highly significant.

#### 3.1.2. Colorectal Cancer

It has been estimated that 1.4 million new cases of colorectal cancer are being annually diagnosed among men and 733,000 among women [35]. For women, colorectal cancer is the third leading cause of cancer death.

As for breast cancer, historically, a lower incidence of colorectal cancer has been observed in Mediterranean countries than other European countries or the USA [36]. Moreover, similarly to breast cancer, a higher adherence to the traditional MedDiet has been proposed as a potential protective factor against colorectal cancer.

In a meta-analysis of prospective cohort studies [44], a higher adherence to the traditional MedDiet was associated with a 14% lower risk of colorectal cancer (HR = 0.86, 95% CI 0.80–0.92).

Unfortunately, to our knowledge, no large prospective cohort studies or large case-control studies have been recently conducted aiming to assess the association between extra-virgin olive oil consumption and the risk of colorectal cancer.

It must be underlined that studying cancer development as related to diet is very difficult to carry out in humans due to the scantiness (or absence) of surrogate markers that dietary interventions could modulate. Therefore, even if we have robust epidemiological studies (with consistent results, good control for confounding and corroborated by a plethora of in vivo investigations) the true nature and extent of olive oil’s contribution to chemoprevention might never be confirmed in humans. Nevertheless, older studies by Machowetz et al. [45] and Salvini et al. [46] performed in healthy males and postmenopausal women, respectively, have reported reduced oxidative DNA damage after short-term use of phenol-rich olive oil, which might translate into a lower cancer risk.

### 3.2. Extra-Virgin Olive Oil and Cardiovascular Disease

Southern European countries have the lowest incidence rates of coronary heart disease [47,48] in spite of a high prevalence of classical cardiovascular risk factors [49,50]. As mentioned above, the near totality of evidence comes from epidemiological studies, with the notable exceptions of the Lyon Heart Study and the PREDIMED randomized trial.

An inverse relationship between olive oil intake and coronary heart disease mortality and incidence has been reported within the frame of EPIC cohorts [39,40,41,51]. The Three-City study also found an inverse relationship between olive oil and stroke in women [52]. In this regard, Guasch-Ferré et al. reported that olive oil—specifically extra-virgin—intake in the context of a MedDiet was associated with a reduction in the risk of CVD and mortality in older high cardiovascular-risk individuals [53].

A recent meta-analysis of 32 cohort studies showed that olive oil, but not MUFAs, consumption was associated with a reduced risk of all-cause mortality, CVD events, and stroke when comparing the upper to the lower tertile of consumption [54]. This finding indicates that the minor constituents of olive oil could be responsible for its health benefits [55,56].

#### Modulation of Classic Cardiovascular Risk Factors

As mentioned, a traditional MedDiet, supplemented with virgin olive oil or nuts, reduced blood pressure and the risk of hypertension in high cardiovascular risk subjects [19,57]. In addition, in the context of the PREDIMED trial, DBP decreased in hypertensive women after both MedDiet interventions [58]. Lower values of DBP in the two groups promoting the MedDiet with extra virgin olive oil or nuts versus the control group were also observed in PREDIMED’s 4 year-follow up [59]. Moreover, the 5-year PREDIMED intervention with unrestricted-calorie MedDiet was associated with a decrease in weight and less gain in central adiposity compared with the control low-fat diet [30]. In this regard, the effect of the MedDiet on weight loss was previously reported in the DIRECT trial, where participants following a calorie-restricted MedDiet achieved a higher weight loss compared to low-fat and low-carbohydrate diets [60,61].

With regards (poly)phenol-rich extra-virgin olive oil, a meta-analysis of 69 experimental studies showed moderate effects for lowering SBP, with no effects on DBP [62]. In agreement, a decrease in SBP after virgin olive oil intake was described in hypertensive and coronary heart disease patients [63].

The benefits of olive oil (poly)phenols on circulating HDL cholesterol were evaluated by the European Food Safety Authority (EFSA) and the conclusion was that evidence was insufficient to establish a cause-effect relationship [64]. In a recent meta-analysis concerning the effects of phenolic compound-rich olive oil on cardiovascular risk factors, no effects were observed with regard to lipid profile. In this regard, the small number (*n* = 8) of included studies should be underscored [62]. It is noteworthy that a number of high quality randomized controlled trials have reported a dose-dependent increment in HDL-cholesterol after virgin olive oil intake [55,56], even though the mechanisms behind these purported effects are still unclear.

The EFSA allowed a health claim with regard to the protection conferred by olive oil phenolic compounds (5 mg/day) against LDL oxidation [65]. In this regard, it has been highlighted that oxidation of the LDL particle might play a role in atherosclerosis onset and progression [66,67], but clear-cut human evidence is lacking, trials of antioxidants have failed to demonstrate benefits [68], and the contribution of oxidized LDL to atherosclerosis is still elusive. Yet, a meta-analysis of 300 experimental studies reported that oxidized LDL concentration decreased after high-phenolic olive oil intake [62]. In the EUROLIVE Study, a randomized cross-over controlled trial in healthy subjects, a linear decrease of in vivo lipid oxidative damage was observed along with the phenolic content of olive oil [56]. In addition, other studies have shown a decrease in the ability of LDL to be oxidized after the consumption of phenolic-rich olive oils [56,63,69,70]. Regarding HDL, recent studies have demonstrated that their function can be more relevant than their concentrations [71]. Hernáez et al. reported a rise of cholesterol efflux after the regular intake of virgin olive oil within the framework of the EUROLIVE Study [70]. In agreement, a traditional MedDiet, especially when supplemented with virgin olive oil, improved HDL function [72]. Interestingly, a recent article concluded that high phenolic extra virgin Olive Oil may improve some cardiovascular risk factors [73].

Olive oil intake contributes to the homeostasis of the thrombogenic profile by improving the production of coagulation factors and biomarkers related to platelet aggregation [74]. Olive oil intake attenuates the pro-thrombotic state in the postprandial phase [75,76,77,78] and this is very likely due to the effect of hydroxytyrosol, as ad-hoc human experiments demonstrated [79]. Moreover, phenolic-rich olive oil intake also improved the postprandial pro-thrombotic state (activated coagulation factor VII, tissue plasminogen activator, tissue factor, plasminogen activator inhibitor type-1, and fibrinogen) in several randomized controlled trials in healthy subjects [80] and in hypercholesterolemic patients [74]. In a long-term randomized crossover trial with olive oil consumption, a decrease in plasma fibrinogen in women with initially high fibrinogen concentrations was also reported [81].

Type 2 diabetes includes chronic and uncontrolled hyperglycemia, which leads to serious injury of nerves and blood vessels [82]. In the ATTICA study, adherence to a MedDiet was related to a proper homeostasis of hydrocarbon metabolism in normoglycemic subjects [83]. In addition, a reduction in type 2 diabetes incidence after an intervention based on a traditional MedDiet rich in extra virgin olive oil in elderly high cardiovascular risk volunteers was observed in the initially non-diabetic type 2 subset of participants in the PREDIMED trial [84].

A significant association between certain dietary patterns and lower risk of type 2 diabetes was reported in several studies included in recent meta-analyses [12,85]. In one of these meta-analyses, Schwingshackl et al. concluded that adherence to the MedDiet reduced the risk of developing type 2 diabetes by 19% [12]. Another systematic review of eight meta-analyses of randomized controlled trials and five randomized controlled trials suggested that a MedDiet pattern can be useful for the prevention and management of type 2 diabetes mellitus and pre-diabetic states [86]. The authors found that adherence to the MedDiet was associated with lower glycated hemoglobin (HbA1c) levels and a better profile of cardiovascular risk factors, as compared with a control diet, such as a low-fat one. Two meta-analyses showed that a higher adherence to MedDiet was linked to a reduction in the risk of future diabetes by 19–23% [86]. Another meta-analysis of cohort studies reported that although diets associated with the prevention of type 2 diabetes may vary in their composition, they share some mutual components, including whole grains, fruit, vegetables, nuts, legumes, protein sources such as white meat and seafood, little or moderate alcohol consumption, and reduced intake of red and processed meats and of sugar-sweetened beverages, and abundant use of healthy oils, notably olive oil [87].

Clinical trials of the effects of olive oil on carbohydrates metabolism are scant. Carnevale et al. recently reported, in impaired fasting glucose patients, that virgin olive oil improved post-prandial glucose probably mediated by incretin up-regulation. In this regard, the highest quintile of olive oil intake in comparison with the lowest one has also been associated with a lower risk of type 2 diabetes mellitus; substituting other types of fats and salad dressings with olive oil has been inversely associated with type 2 diabetes mellitus onset [88]. In a cross-sectional study, insulin resistance was found to be lower in individuals who consumed olive oil versus sunflower oil or a mixture of vegetable oils [89]. In particular, a crossover randomized trial reported that a daily intake of extra virgin olive oil (25 mL/day) for eight weeks was inversely associated with fasting plasma glucose and HbA1c, and a number of circulating inflammatory adipokines (such as visfatin), in overweight patients with type 2 diabetes [90]. The PREDIMED trial recently reported that the random allocation of diabetics to a MedDiet with extra virgin olive oil was associated with a significant reduction in the need of starting glucose-lowering medication (Relative risk: 0.78, 95% CI, 0.62 to 0.98) [91].

## 4. Extra-Virgin Olive Oil and Health: Molecular Mechanisms

### 4.1. Cancer

Following the numerous epidemiological reports that show reverse associations between olive oil use and cancer risk, several investigators undertook mechanistic studies to substantiate this interesting hypothesis. Although the provision of antioxidants actually increases cancer risk, most research is being focusing on oxidative stress (now called redox code [92]), whose exact role in cancer etiology is, however, still elusive. Yet, modulation of the redox code by plants’ secondary metabolites might play some roles in cancer and cancer stem cells progression, as well as therapy [93]. Over the last few years, many in vitro and animal studies have demonstrated that olive oil phenolics (alcohols and secoiridoids) possess anticarcinogenic properties, which are likely not mediated by direct antioxidant actions. Several mechanisms have been investigated, including inhibition of angiogenesis [94], of proliferation and invasion [95,96,97,98], induction of apoptosis [99,100], and anti-inflammatory actions [101]. It is likely that if olive (poly)phenols have chemopreventive properties, these are due to a variety of molecular actions rather than to a single one. One example is that of oleocanthal (OC), which was shown as cytotoxic to human melanoma cells, but not to normal dermal fibroblasts; its molecular actions include the inhibition of ERK1/2 and AKT phosphorylation and downregulation of Bcl-2 expression [102]. Of note, OC inhibits cell growth more effectively than classic pharmaceutical COX inhibitors; in addition, OC inhibits colony formation and induces apoptosis-by PARP cleavage, activation of caspases 3/7, and chromatin condensation—in hepatocellular carcinoma (HCC) and colorectal cancer (CRC) cells. Yet, OC is not toxic to primary normal human hepatocytes. In addition, despite being known as an antioxidant molecule, OC induces DNA damage, dose-dependently increases intracellular ROS production, and causes mitochondrial depolarization [103]. Finally, OC has the potential to inhibit the growth of hormone-dependent breast cancer and improve sensitivity to tamoxifen therapy [104].

Oleuropein (OL) also inhibits HepG_2_ (human hepatoma) viability via the induction of apoptosis (i.e., upregulation of BAX and downregulation of Bcl-2), activation of the caspase pathway, and modulation of the phosphatidylinositol 3 kinase/protein kinase B (PI3K/AKT) signaling pathway, in turn suppressing the expression of activated AKT [105]. Of note, a combination of OLE and cisplatin shows synergistic effects, i.e., as compared with the addition of individual molecules effects in HepG2, leading to an increase of NO and of the pro-nerve growth factor (NGF)/NGF ratio. This is accompanied by a dose-dependent upregulation of caspase-3 and a concomitant downregulation of MMP-7 gene expression [105].

With regards hydroxytyrosol (HT), some papers reported chemoprevention via prevention of DNA damage in PBMC and inhibition of breast (MDA and MCF-7), prostate (LNCap and PC3) and colon (SW480 and HCT116) cancer cell proliferation [106]. HT also reduces papillary (TPC-1, FB-2) and follicular (WRO) thyroid cancer cell proliferation and viability by promoting apoptotic cell death via intrinsic pathways. However, these experiments were performed with high doses of HT, which diminishes their physiological relevance [107]. HT and two of its colonic metabolites (phenylacetic and hydroxyphenylpropionic acids) are able to arrest the cell cycle and promote apoptosis in HT-29 and Caco-2 cells [108]. Oleacin, another phenolic compound found in EVOO, has been described to inhibit tumor-initiating cells by metabolic and epigenetic mechanisms using concentrations close to physiologic metabolism [109,110,111].

Cellular senescence, which impairs the proliferation of damaged or premalignant cells, also plays a role in aging and age-related diseases (including cancer) and is an interesting therapeutic target [112]. Indeed, modulation of the senescence-associated inflammatory phenotype has been recently suggested as an important mechanism of action of olive oil phenols. In a recent study performed in pre-senescent human lung (MRC5) and neonatal human dermal (NHDF) fibroblasts, a four-to-six weeks supplementation with 1 μM HT or 10 μM OLE aglycone (OLE) prevented TNFα-induced inflammation, decreased the number of β-galactosidase-positive cells and p16 protein expression, IL-6, metalloprotease secretion, COX-2, and α-smooth-actin levels. In NHDF, OLE and HT treatment counteracted the senescence-associated increases of cyclooxygenase 2 (COX2) expression, nuclear transcription factor-kappa B (NFκB) protein level, and nuclear localization [113].

In short, an imbalance of the redox code increased inflammation, and deregulation of cell cycle concur to alter cellular replication [114]. Major players include the activation of some transcription factors, such as NF-κB, signal transducer and activator of transcription 3 (STAT3), MAPK, and the hypoxia-inducible factor 1α (HIF1α). These transcription factors dictate the production of inflammatory mediators, e.g., cytokines and chemokines and the activation of COX2, in turn recruiting and activating leukocytes and triggering the inflammasome of tumor cells, resulting in more inflammatory mediators being produced and a cancer-related inflammatory microenvironment being generated and propagated in a vicious cycle [79]. Therefore, we can hypothesize that olive (poly)phenols would primarily act as anti-inflammatory molecules, but their manifold actions on cell signaling could also contribute to chemoprevention.

One important cautionary note concerns the exceedingly high concentrations of (poly)phenols that are frequently employed in in vitro studies. Due to the low bioavailability of such compounds, extrapolation of in vitro data to human physiopathology is often questionable. In this respect, the most appropriate “high-concentration” studies are those carried out on GI tract cells. Other data should be interpreted in light of this caveat.

In summary, olive oil and its (poly)phenols might play important roles in lessening cancer risk, as observed in the Mediterranean area [115]. Whether the association between olive oil consumption and chemoprevention is causal or casual is worth further investigation, but—even though human trials are impractical—the advice to consume olive oil as the predominant source of fat to lower cancer risk rests on solid bases.

### 4.2. Cardiovascular Disease

The molecular and cellular mechanisms underlying the cardioprotective effects of olive oil and its components are manifold [116,117]. Some of them are discussed below.

#### 4.2.1. Extra Virgin Olive Oil (EVOO) and Atherosclerosis

Atherosclerosis is a common finding in cardiovascular diseases. In a subcohort of the PREDIMED that evaluated intima-media thickness (IMT), an inverse association between extra virgin olive oil (EVOO) consumption and IMT was described [118], suggesting a protective role of olive oil against the development of carotid atherosclerosis in persons at high cardiovascular risk [119]. In animal models, the administration of EVOO and some of its components such as hydroxytyrosol and squalene [120] reduced atherosclerotic lesions [121,122]. Recently, the EVOO secoiridoid oleacein was found to reduce secretion of metalloproteinases from carotid plaques [123]. Undoubtedly, this area needs to be expanded with new image technology to solve some current controversies.

#### 4.2.2. EVOO and Endothelial Dysfunction

Endothelial dysfunction plays a key role in the development of atherosclerotic cardiovascular disease. The consumption of a MedDiet rich in extra-virgin olive oil improved endothelial function compared to the same yet low-fat diet in patients with prediabetes and diabetes participating in the CORDIOPREV clinical trial [124]. Australian men and women consuming a MedDiet rich in extra-virgin olive oil for 6 months showed significantly lower SBP and improved endothelial function [125]. Lower values of DBP were found in the PREDIMED group receiving EVOO [59].

In hypertensive rats, administration of EVOO slowed down the age-dependent increase in systolic blood pressure with concomitant decreases in nitric oxide (NO) and 8-isoprostane [126]. This beneficial effect has been attributed to the phenolic compounds present in virgin olive oil, since these compounds reverted the decreased endothelial NO synthase phosphorylation, and consequently intracellular NO levels, and increased endothelin-1 synthesis in ECV304 incubated with high glucose and fatty acids [127]. However, in Wistar rats fed extra-virgin olive oil with or without phenolic compounds, aorta vascular endothelial adhesion molecule-1 and E-selectin were lower compared to control animals. These results suggest that these changes are independent of phenolic compounds [128].

#### 4.2.3. EVOO and Transcriptomics

Acute intake of high phenolic EVOO was able to modify the transcriptome of peripheral blood mononuclear cells through the modulation of different pathways associated with the pathophysiology of cardio-metabolic disease. These beneficial effects were more pronounced in healthy subjects who used high phenolic EVOO [129], thus providing further support to notion that olive phenolic compounds could be employed to treat chronic inflammatory states [130].

#### 4.2.4. EVOO and Plasma Lipids

When 91 healthy men and women were randomized to consume 50 g daily of extra virgin coconut oil, EVOO, or unsalted butter for four weeks, the latter significantly increased total cholesterol/HDL-C ratio and non-HDL-C compared with coconut oil. Coconut oil and EVOO use lead to similar results for both parameters. These results evidence the complex mixture that virgin oils obtained from fruits represent [131]. In a prospective longitudinal and comparative study where 18 postmenopausal women participated in two periods of dietary intervention with either butter or extra virgin olive oil (28 days each), the latter decreased total/HDL-cholesterol and triglycerides/HDL-cholesterol [132].

The effects of corn oil (CO) versus extra-virgin olive oil intake was tested in 54 men and women consuming 54 g of either oil for 21 days in a randomized double-blind controlled-feeding crossover trial. CO reduced non-high-density lipoprotein cholesterol compared to EVOO. There were no differences on HDL-cholesterol but APOA1 increased more with EVOO compared with CO intake [133].

The post-prandial 2-h lipid profile of 25 healthy subjects randomly allocated (in a cross-over design) to Mediterranean-type meal with 10 g of either EVOO or CO showed a significantly lower increase of LDL-C and ox-LDL in the EVOO group compared with the CO one [134]. In a random sub-sample of individuals from the PREDIMED study, after 1-year of intervention, the group consuming the MedDiet enriched in EVOO showed increased LDL resistance against oxidation and decreased the degree of LDL oxidative modifications compared to the low-fat group [135]. In the same study, the branch receiving EVOO showed increased cholesterol efflux capacity, decreased cholesteryl ester transfer protein activity and increased HDL ability to esterify cholesterol, paraoxonase-1 arylesterase activity, and HDL vasodilatory capacity, resulting in a more functional HDL [72]. HDL cholesterol efflux capacity was higher in 47 healthy European male volunteers consuming 25 mL/d EVOO of high phenolic content compared to when the same subjects received a phenolic—poor EVOO for 3 weeks [70]. Likewise, in apolipoprotein-E-deficient mice, 7 µL/mouse/day of EVOO for 2 months stimulated cholesterol efflux rate from mouse peritoneal macrophages [121]. Overall, these findings propose a better status of plasma lipoproteins by the administration of EVOO.

According to lipidomics analyses, plasma ceramide levels decreased in participants receiving the MedDiet, including EVOO in the PREDIMED trial [136]. The intervention of EVOO significantly changed 20 lipid species compared with the control group. However, the latter changes were not associated with subsequent CVD risk [137] and the true contribution of such modifications to the observed risk reduction requires further investigations.

In summary, the effects of EVOO on plasma lipids are yet to be fully elucidated and can only be evaluated in substitution/replacements scenarios. This is true for all kinds of lipid trials and data should be interpreted in light of these limitations.

#### 4.2.5. EVOO and Adipose Tissue Metabolism

The adipose tissue is one of the targets of cardiometabolic prevention because inflammation, maybe the elusive oxidative stress, and secretion of adipocytokines contribute to cardiovascular risk. Much research has devoted to the study of phenolic compounds in these cells. In this regard, in 3T3-L1 adipocytes, HT was able to promote mitochondrial function by stimulating mitochondrial biogenesis through increasing the promoter transcriptional activation and protein expression of peroxisome proliferator-activated receptor coactivator 1 alpha [138]. HT, in nutritionally relevant amounts, was also able to augment the glutathione-driven antioxidant enzymatic pathways in the adipose tissue [139]. A summary of molecular and cellular mechanism of CVD is included in Table 1.

### 4.3. Autoimmune Diseases/Immune-Inflammatory Diseases

Immune cell plasticity is mainly involved in the pathogenesis and resolution of chronic inflammatory autoimmune processes. Dietary components, especially dietary fatty acids and (poly)phenols, modulate the immune response and might be exploited as a pharma-nutritional methodology for the prevention and management of these illnesses [140].

EVOO administration in lipid emulsions might be beneficial to immunocompromised patients, as it may contribute to the reduction of typical inflammatory activity from autoimmune disorders without exacerbating the susceptibility to pathogens [141]. In this context, the effects of EVOO have usually been ascribed to its apolar lipids or to oleic acid [142,143,144], even though there is no consensus on this [145,146]. Indeed, EVOO (poly)phenols possess anti-inflammatory and immune-regulatory effects, devoid of the typical effects of classical pharmacotherapy [147]. This notion triggered research on EVOO and its bioactive components as an alternative nutritional therapeutic strategy for immune-inflammatory diseases such as inflammatory bowel disease (IBD), rheumatoid arthritis (RA), systemic lupus erythematosus (SLE) and sclerosis.

#### 4.3.1. Inflammatory Bowel Disease (IBD)

IBD, comprising of Crohn’s disease, ulcerative colitis, and an unclassified type of IBD are characterized by chronic and recurring inflammation of the intestinal mucosa. The etiology of IBD is weakly understood, but environmental factors, infectious diseases, ethnicity, and genetic susceptibility may be implicated [148]. It has been hypothesized that aberrant immune response with an uncontrolled T cell activity to intestinal microorganisms in genetically predisposed individuals may be involved in the etiopathogenesis of IBD [149]. Recent reports indicate that dietary factors might affect the risk of progressing IBD; in addition, dysbiosis caused by nutrition plays a role to the pathogenesis of IBD, thus, diet may contribute as a symptomatic treatment for irritable bowel syndrome-like symptoms in IBD [150]. Accordingly, nutritional strategies focusing on adjusting the ratio of consumed nutrients that are pro-inflammatory or anti-inflammatory have been explored as primary or adjunct therapies for IBD [151]. Low-fat diets seem to be mostly helpful. In addition, some lipid sources, such as EVOO, might have a therapeutic effect [152].

In agreement with the literature, experimental murine models of intestinal inflammation have confirmed the protective effects of dietary EVOO supplementation on DSS-induced chronic colitis through peroxisome proliferator-activated receptor gamma (PPAR-γ) up-regulation and mitogen activated protein kinases (MAPKs)/nuclear transcription factor-kappa B (NF-κB) signaling pathways inhibition, decreasing the inflammatory cascade [153]. The EVOO diet induced significant protective effects in chronic DSS-induced colitis by decreasing cyclooxygenase (COX)-2 and inducible nitric oxide synthase (iNOS) overexpression in addition to cytokine modulation via downregulation of p38 MAPK and therefore, is taken into consideration as a beneficial functional food for ulcerative colitis management.

Supplementation EVOO’s (poly)phenols [154] such as HT and its acetate (HT-Ac) improved experimental colitis [153,155] via reducing upregulation of both pro-inflammatory enzymes COX-2 and iNOS. In addition, HT-Ac inhibited the NF-κB signaling pathway in addition to JNK MAPK phosphorylation [156]. Squalene also lessens signs of inflammation in the gut mucosa, probably via downregulation of p38 MAPK and NF-κB signaling pathways [156].

EVOO’s minor components have also been evaluated ex-vivo, in blood and intestinal T cells from IBD patients enrolled with active disease or in remission, as well as in healthy donors. Importantly, these molecules promoted apoptosis and attenuated the activation of both intestinal and blood T cells from IBD patients, reducing the frequencies of activated CD4^+^T cells and CD8^+^ T cells, identified by the expression of CD69 and CD25 in blood and intestinal mucosa. Likewise, the unsaponifiable fraction modulated gut homing properties of T cells, decreasing the expression of integrin β7^+^ on blood T cells from IBD patients [157].

Finally, Sánchez-Fidalgo and colleagues demonstrated that EVOO-enriched diets had protective/preventive effects on the ulcerative colitis (UC)-associated colorectal cancer (CRC). This beneficial effect was associated with an improvement of the disease activity index and a smaller number of dysplastic lesions, showing that the EVOO diet, in addition to inhibiting DSS-induced chronic inflammation, could also reverse the precancerous state induced by DSS in mice [158].

#### 4.3.2. Rheumatoid Arthritis (RA)

RA is a long-term progressive multistep and complex process that leads to immune inflammation which involves numerous joints, inducing painful swelling and joint stiffness, accompanied with cartilage damage and bone erosion, leading finally to joint destruction [159,160]. In the pathogenesis of RA, the following are involved: activated innate and adoptive immune cells, resident cells such as ostecoclasts, fibroblast-like synoviocytes and chondrocytes, as well as autoantibodies such as rheumatoid factor and anti-citrullinates peptide antibodies [161].

In the inflammatory process that characterizes RA, several cell types and cytokines are involved. As an example, activated T cells distinguish into diverse T helper cell subsets with different cytokine profiles and effector functions, which leads to autoantibody generation and a further secretion of cytokines, in turn generating articular and extra-articular symptoms. Of note, activated T cells release interferon (IFN)-γ, interleukin (IL)-2, IL-12, IL-18, and tumor necrosis factor (TNF)-α. Furthermore, such molecules activate macrophages to secrete other pro-inflammatory cytokines, induce the differentiation of B cells and activate the release of MMPs [162]. The objective of conventional pharmacological therapy is to finish off or reverse cartilage destruction and diminish the pain devoid of untoward effects. However, current treatments are not efficient in all patients and possess a number of disadvantages, such as high cost, necessity for parenteral administration and potential adverse effects. Consequently, nutritional therapy as complementary and alternative medicine is under development as an innovative strategy in RA management.

In connection with this, Rosillo and colleagues have deeply investigated the effects of EVOO and its phenolic compounds in RA. In a mouse model of collagen-induced arthritis (CIA), they found that EVOO dietary (EVOO diet for 6 weeks) [163] and EVOO-PE (100 and 200 mg/kg, for 13 days) [164] consumption prevented RA development, reducing joint edema and cartilage destruction. These effects were associated with a reduction of pro-inflammatory cytokines secretion, such as TNF-α, IL-1β, IL-6 and IL-17 in comparison with the control CIA group and minimized serum levels of cartilage oligomeric matrix protein (COMP) and MMP-3. These immuno-modulatory effects of EVOO and its phenolic compounds could be related to the stimulation of the nuclear factor (erythroid derived 2)-like the 2 (Nrf2)/heme oxygenase (HO-1) pathway and the inhibition of the relevant signaling pathways, for instance, NF-κB, signal transducer and activator of transcription 3 (STAT3) and MAPKs, which control the inflammatory response.

Preclinical studies have also confirmed that isolated EVOO (poly)phenols, such as HT, oleuropein (OL) and oleocanthal, have anti-inflammatory and anti-arthritic properties. For example, Oleocanthal reduced IL-6 and macrophage inflammatory protein (MIP)-1α at the protein and mRNA levels and decreased both LPS-induced iNOS protein expression and NO production when added to lipopolysaccharide (LPS)-stimulated ATDC-5 murine chondrocytes [165,166].

Likewise, OL-aglycone improved clinical markers and histological status in joints and paws on CIA mice model. In this model, OL-aglycone reduced the plasma levels of pro-inflammatory cytokines (TNF-α, IL-1β and IL-6), chemokines (MIP-1α and MIP-2) and myeloperoxidase (MPO) activity as index of neutrophil infiltration [167]. In vitro OL decreased IL-6, TNF-α, MMP-1 MMP-3 and COX-2 overexpression in IL-1β-stimulated human synovial fibroblasts [168]. Furthermore, HT-supplemented refined olive oil had protective effects in a CIA rat model by diminishing paw edema and COX-2 and iNOS expression, as well as bone resorption, soft tissue swelling and osteophyte formation. Possibly, the beneficial effects could speculatively be ascribed to the potential synergism of the HT and the MUFA content of the olive oil [169]. Moreover, an HT-Ac enriched diet showed prophylactic effects, improving the arthritic process in the CIA mice model, ameliorating the arthritis score and blocking the migration of inflammatory cells, joint edema, synovial hyperplasia and cartilage erosion. Furthermore, the HT-Ac diet reduced serum synovial and cartilage biomarkers COMP and MMP-3 via the activation of the Nrf2/HO-1 pathway and the inhibition of JAK-STAT, MAPKs and NF-κB signaling pathways [170]. Conversely, HT did not show any positive effect in this model. The different behavior of the polyphenol could be due to the higher lipophilicity of Hy-Ac compared with HT because of the presence of an ester group [171,172], resulting in a better absorption and bioavailability [173]. On the other hand, in vitro studies have demonstrated the anti-inflammatory effect of HT and HT-Ac in human IL-1β-stimulated SW982 cells, which was characterized by a significant reduction of MMP-1, MMP-3, COX-2, mPGES-1 and cytokines IL-6 and TNF-α, which could be possibly related via MAPKs and NF-κB signaling pathways inhibition [174].

#### 4.3.3. Systemic Lupus Erythematosus (SLE)

An autoimmune and chronic inflammatory disease is currently being actively investigated is SLE. This disease could affect numerous organ systems such as the kidneys, skin, brain, and joints, among others, and it is typified by a deposition of autoantibodies and immune complexes, leading to extensive tissue damage and numerous complications, which influence quality of life [175]. In the etiopathogenesis of SLE genetic, epigenetic and environmental factors, as well as nutrition and infection, concur [176]. Immune complexes, autoantibodies, imbalance of T-helper cell subsets (TH1/TH2/TH17) and regulatory T-cells (Tregs), play an important role in SLE tissue damage. In the course of their active phase, higher serum levels of IFN-γ, TNF-α, IL-4, IL-6, IL-10, IL-12, IL-17 and IL-18 and lower levels of IL-2 are found. Consequently, the immune response is amplified through the clonal expression of B-lymphocytes, producing an inflammatory reaction via TLRs, accompanied by a release by lipid mediators, cytokines and neutrophil infiltration [177].

The unknown etiology of SLE makes it difficult to develop therapeutic strategies aimed at fighting this multifaceted disorder [178]. However, recently biological drugs have gained more attention than conventional drugs in the SLE treatment [179]. Targeting specific cytokines (e.g., IL-6, IL-17, IL-23, and IFN-γ) and downstream regulatory molecules (JAK/STAT pathways), reducing circulating B cells and autoantibody titers [178,179,180], as well as modulating IL-27 [181] and IL-35 [182] appear to be interesting therapeutic routes to hinder SLE.

Moreover, diet quality in SLE patients is relevant since these patients suffer a higher risk of other pathologies which are directly influenced by diet. Therefore, nutritional therapy by means of diet changes and the use of nutritional supplements could be a promising tool for SLE due to the potential prophylactic effects without the adverse effects of the traditional pharmacological therapy, possibly reducing comorbidities and improving quality of life in SLE patients [183].

In this context, EVOO included in MedDiet have shown immune-modulatory effects, brought to light their possible supportive role in primary and secondary prevention and management of SLE. Recently, Aparicio-Soto and colleagues evaluated the possible beneficial effects of an EVOO diet and its phenolic compounds HT and HT-Ac in a pristane-induced SLE model in mice. After pristane-SLE induction for 24 weeks, an EVOO supplemented diet [147] and dietary HT and HT-Ac supplementation [184] notably ameliorated paw swelling and proteinuria levels and improved histological renal damage, diminishing kidney abnormalities like inflammatory mononuclear cells in the renal interstitium or “thyroidization” in scattered renal tubules. Moreover, the EVOO, HT and HT-Ac diets decreased MMP-3 serum and PGE2 kidney levels, in addition to IL-6, IL-10, IL-17 and TNF-α production in splenocytes, possibly via up-regulation of Nrf-2/HO-1 and inhibition of JAK/STAT, MAPK and NF-κB signaling pathways.

The results obtained with PBMC from inactive SLE patients confirm further evidence on the immunomodulatory role of EVOO polyphenols in humans. In a recent study, PBMCs stimulated with phytohemaglutinin and incubated for 24 h with PE showed a decreased activation of T cells, reduced the release of IFN-γ, TNF-α, IL-6 and IL-10, together with the inhibition of NF-κB and ERK. Of note, the observation that PE reduced IL-10 in SLE patients and increased IL-10 levels in healthy subjects highlighted the potent ability of phenolic compounds to regulate IL-10 deregulation [184].

#### 4.3.4. Sclerosis

Amyotrophic lateral sclerosis (ALS) is a progressive adult-onset neurodegenerative disease characterized by a brain and spinal cord specific loss of motor neurons. ALS leads to weakness and paralysis due to a progressive muscle atrophy, and finally, death because of the compromise of respiratory muscles. In ALS patients and ALS murine models, specific immune abnormalities and inflammatory markers are found in the central nervous system and blood. Moreover, it is speculated that the immune response could regulate the progression of the disease, but it can also play a protective role. Currently, only a palliative ALS treatment is in use, but strategies that improve protective immunity are just now in progress [185]. In this framework, a diet enriched with EVOO improved pathological findings and deferred the disease onset in an ALS model with transgenic mice that carry a mutant form of the antioxidant enzyme copper/zinc (Cu/Zn)-superoxide dismutase 1 (SOD1) (SOD1G93A variant). Additionally, the EVOO diet increased survival, motor performance and muscle fibre areas in comparison to mice fed a palm oil diet. Likewise, an improvement of the muscle status by reducing the expression of myogenic factors (MYOD1 and MYOG), autophagy markers and endoplasmic reticulum stress markers, such as transcription factor 6 (ATF6) and 78 kDa glucose-regulated protein (Grp78) [183], were observed in animals fed the EVOO-enriched diet [186].

Furthermore, in motoneurons/glia co-cultures obtained from wild type or SOD1 mutated (SOD1 G93A) mice, polyphenols extracts (PE) avoided cell death and blocked NO induced production, either after LPS stimulation or because of the presence of SOD1 mutation, suggesting that PE could show neuroprotective effects probably modulating inflammatory mediators [187].

Multiple sclerosis (MS) is a chronic autoimmune disease of the central nervous system (CNS) characterized by demyelination and axonal degeneration. It is an inflammatory disease caused by the interaction between genetic and environmental risk factors with relapsing-remitting or progressive course [188]. Immune system deregulation, which involves T and B cells, chronic inflammation with activation of microglia, enhancing mitochondrial damage, in addition to oxidative stress and membrane channel dysfunction, are important hallmark features implicated in MS pathogenesis [189].

To date, current therapeutic options for MS remain disappointing and challenging, since they only aim to minimize symptoms and, if possible, improve function. However conventional therapeutic options present multiple undesirable side effects. Therefore, highlighting emerging therapeutic strategies is needed.

Nevertheless, to study MS, several different models of MS exist. The experimental autoimmune encephalomyelitis (EAE) model is the best understood and most commonly used [190]. Natural triterpenes present in EVOO, such as oleanolic acid (OA) and erythrodiol, display potent anti-inflammatory, hepatoprotective, anti-hyperlipidemic and immunomodulatory activities. In this sense, the administration of OA before or at the early onset of the disease decreased neurological signs in mice, decreasing induced levels of osteopontin and ICAM-1 in CNS tissue, accompanied by a reduction of blood-brain barrier leakage and lower infiltration of inflammatory cells within the CNS. In addition, OA could exhibit a modulator role in Th1/Th2 polarization, decreasing pro-inflammatory cytokines and chemokines serum levels. In addition, EAE-animals that were treated with OA showed lower serum immunoglobulin (Ig) G, IgG1 and IgG2a compared with untreated EAE-mice [191].

In a similar study using the same experimental model, treatments with OA or erythrodiol showed preventive effects, preventing blood-brain barrier disruption and blocking infiltration of inflammatory cells into the CNS by preventing up-regulation of specific serum myelin oligodendrocyte glycoprotein, IgM antibodies and switched cytokine production towards a Th2/regulatory profile, diminishing Th1 and Th17 cytokines levels and augmenting the release of Th2 cytokines in both serum and spinal cord. In addition, both compounds affected the humoral response causing auto-antibody production inhibition. In vitro, by the addition of inflammatory stimuli to microglia, triterpenes inhibited ERK and rS6 phosphorylation and reduced the synthesis of proinflammatory mediators. Thus, triterpene therapy might be a helpful tool of clinical interest for human autoimmune and neurodegenerative diseases, including MS and other Th1 cell-mediated inflammatory diseases [192].

Astrocytes, the main neural glial cell type, play a dual role in modulating neuronal activity against oxidative stress but pathogenic stimuli may disturb astrocytic function stimulating the microglia in inflammatory processes, thus compromising neuronal functionality and viability in MS [193]. In this regard, in LPS-activated primary rat astrocytes HT in an olive oil extract (Oliplus^©^) reduced dose-dependently the expression and activity of the enzyme’s gelatinases A (MMP-2) and B (MMP-9). By contrast, Oliplus was only partially effective in inhibit MMP-2 and MMP-9 activities in serum samples from MS patients, suggesting that HT could exert inhibitory effects on MMPs activity and may represent a powerful tool to improve the wellness of MS patients [194].

## 5. The Sustainability Issue: The Case for Olive Oil

Olive-based products are important contributors to the agricultural economy of the European Union (EU)’s Southern countries, with about 5 million hectares (ha) of plantations and a production value of over 7000 million Euros every year. The EU is the world’s largest olive oil producer, accounting for 70% to 75% of world production and more than one third of table olives. In 2016, 74% of the EU’s olive oil was produced in Spain, where more than half of the EU’s surface of olive plantations is found, while a further 22% was almost equally produced between Greece and Italy. Economic forecasts point to increased EU production (especially in Spain, where a 10% increase is projected by 2026, and Portugal) and increased demand from non-producing countries, which will enhance the EU’s leading role on the export market. In terms of international trade, exports are predicted to grow over 45% by 2026. These expectations will require increasing the size of plantations and introducing mechanization accompanied by innovative harvesting solutions, new cultivars, and pest management to increase profits [195].

### 5.1. Use of Pesticides to Protect Olive Trees from Harmful Pests

Insects, weeds, and pathogens can reduce crop production by 25%–50%. Therefore, synthetic pesticides are used to protect plants or plant products against harmful organisms or to prevent the action of such organisms. Pest control increases the global volume of food production [196].

Pesticides are marketed as plant protection products (PPP), which are commercial formulations containing active substances, safeners, or synergists. Furthermore, pesticides are also used for public health, as they are intended to limit the potential for vector-borne diseases. About 3.5 billion kilograms of pesticides are worldwide used each year. Although these chemicals improve crops health and production, their continued application can lead to pest resistances and human and environmental health impairment [196,197]. Hence, new approaches are being considered for a sustainable agriculture to meet the growing global food demand while preserving human and environmental health.

Olive groves constantly need effective care and treatment to avoid certain diseases or pest attacks that weaken the trees, making the olives not suitable for human consumption or reducing their desired quality. Moreover, recent climatic conditions and the olive fly have affected olive yields [198]. Due to these reasons, PPP are widely used for olive grove to ensure crop production and harvest yield. However, it is important to know what and when PPP have to be applied to olive trees to obtain a quality fruit with an optimal quantitative yield. Common fungicides used for olive grove include copper oxychloride and tebuconazol. Other PPP commonly used are insecticides (i.e., lamda-cyalothrin, deltamethrin, dimethoate, phosmet and spinosad) and herbicides (i.e., glyphosate, flazasulfuron and other sulfonylurea compounds) [199]. The overuse of these chemicals can threaten the human health if they are transferred to olive oil, especially if the recommended doses and the application intervals are not respected.

### 5.2. Pesticides as Regulated Substances

Plant protection and biocidal products (both covered under the term “pesticides”) are used in agriculture in order to secure yield and food safety in plant production and animal husbandry. However, since pesticides may have an impact on the environment and non-target organisms, including animals and humans, they are regulated and assessed for pre-market approval in many countries.

The EU regulatory framework is based on the Regulation (EC) No 1107/2009 of the European Parliament and of the Council concerning the placing of PPP on the market. This regulation is intended to guarantee a high degree of protection of human, animal and environmental health, as well as to safeguard the competitiveness of community agriculture. This regulation specifically protects vulnerable population groups such as pregnant women, infants and children. Furthermore, under this regulation, the Commission is required to identify active substances with certain properties as candidates for substitution. This process will promote the use of less harmful pesticides and provide incentives for industry to develop pesticides with less hazardous properties. Apart from the active pesticide ingredients, commercial PPP contain adjuvants and synergists which modify the uptake and potency of the active substances. Since these chemicals can be toxic to both humans and the environment, the Regulation (EC) No 1107/2009 also lays down rules relative to adjuvants, co-formulants, protectors and synergists.

Regulation (EC) No 396/2005 of the European Parliament and of the Council lay down the maximum residue limits (MRLs) for pesticides in food and feed of plant and animal origin to ensure a high degree of protection to all EU consumers, including the most vulnerable ones (such as children, embryos and fetuses). This regulation replaced the different national MRLs by a single harmonized MRL at the EU level, thus guaranteeing free trade across European countries. This regulation also guarantees that pesticide residues are not present at levels that pose an unacceptable risk to animals. When setting MRLs, the cumulative and synergistic effects of those pesticides having a common mechanism of toxicity is also considered.

Another relevant piece of legislation is Directive 2009/128/EC of the European Parliament and of the Council, which establish a framework for community action to achieve the sustainable use of pesticides by reducing the risks and effects of the use of pesticides on human and environmental health. Likewise, the promotion of integrated pest management and non-chemical alternative approaches or techniques to pesticides is encouraged. However, total sales of pesticides across the EU, either as a whole or disaggregated by groups of pesticides, remained constant over the period 2011–2015, with around 400,000 tones. The stable EU demand for pesticides indicates that the risks of pesticides to humans and the environment have remained constant despite the implementation of the National Action Plans under the Directive on the Sustainable Use of Pesticides [200].

### 5.3. Pesticides and Health Risks for Humans and the Environment

Because of their biological activity, pesticides are intended to be hazardous for unwanted organisms. However, their lack of selectivity is a matter of concern as most pesticides act by interfering with molecular processes that are common to a wide range of target organisms (pests, weeds, and fungi) and non-target organisms (humans and wildlife) [197]. In addition, chemical pesticides contribute to environmental pollution, development of resistance in target insects, decreased biodiversity, and outbreaks of secondary pests that are normally controlled by natural enemies [201]. Accordingly, over time, pesticides have generally become less persistent and more species-specific, thus reducing their environmental impact.

The effects of pesticides on human health depend on both the toxicity of the pesticide (or mixture of pesticides) and the magnitude and duration of exposure. Farm workers, particularly pesticide sprayers, experience the greatest exposure through the skin, followed by populations living near the sprayed areas. Likewise, the general population is exposed to pesticide residues present in food or water (or from residential pesticide use) which might pose a risk for consumers’ health [202].

While developing countries use only 20% of the world’s pesticides, they suffer 99% of deaths from pesticide poisoning, which amounts to 200,000 acute poisoning deaths each year [203]. This observation may be partially accounted for the use of highly toxic pesticides. For instance, around 10% of pesticides used in Africa belong to the World Health Organization Class 1a (extremely hazardous) and 1b (highly hazardous), which have mostly been banned in developed countries [197].

The acute and chronic toxicity of a pesticide is determined by exposing experimental animals to a wide range of single or repeated doses over a period of time, following test guidelines to fulfill the pesticide registration data requirements. In humans, non-fatal acute health effects consist of episodes of dizziness, headaches, nausea, vomiting, blurred vision, skin and eye irritation. In contrast, human health effects related to chronic pesticide exposure remain unclear because many studies do not properly assess the time and extent of exposure to these compounds. In 2013, EFSA published an External Scientific Report [204] based on a systematic review of epidemiological studies on the association between pesticide exposure and 23 major categories of human health outcomes. The most consistent evidence was found for Parkinson’s disease and childhood leukemia, thus supporting previous meta-analyses. Moreover, an increased risk was also found for diverse health outcomes less well studied to date, such as liver cancer, breast cancer, stomach cancer, amyotrophic lateral sclerosis, asthma and type II diabetes. Although increased risks were observed as well for other outcomes, such as endocrine disorders, asthma and allergies, diabetes and obesity, these findings warrant further exploration [204].

As insecticides and herbicides are sprayed or spread across entire agricultural fields, over 95% of these compounds has been estimated to reach destinations other than their target species, often resulting in harm to non-target wildlife and ecosystems disruption [205]. Pesticide drift is the airborne movement of droplets or particles which are carried by wind to other crop fields, pasture fields, or human residences, potentially affecting off-target species. Liquid pesticides applied to crops can also volatilize and be blown by wind into nearby areas, thus threatening plants, animals and the environment. Weather conditions at the time of application along with temperature and relative humidity influence the spread of the pesticide in the air [206,207].

Pesticides applied to crops can enter soil, surface water or groundwater via leaching and run-off, which may negatively affect non-target species in both terrestrial and aquatic ecosystems. This impacts habitat function and contributes to biodiversity loss, including large reductions of insect populations [200]. The major routes through which pesticides reach the water include runoff from treated plants and soil, drift outside of the intended area when sprayed, percolation or leaching through the soil, and accidental spill. Water contamination by pesticides depends on their solubility, distance from the application site to a body of water, weather, soil type, presence of a growing crop, and the method used to apply the chemical [207]. Pesticides in European streams have been linked to a reduction in regional biodiversity by up to 42% for invertebrates [208]. This reduces the quality of ecosystem services and the provision of clean drinking water [200].

The use of pesticides decreases the soil quality and reduces its content in organic matter, thus decreasing the potential for water retention. The fate of pesticides in soils is influenced by their sorption, degradation and movement, which are the main drivers of the persistence of pesticides in soil. The rate and amount of pesticides sorption, degradation and leaching in soil depend on the chemical nature of pesticide and soil properties. Sorption affects the bioavailability of pesticides and depends on the content of organic matter in the soil. Bioactivity, persistence, biodegradability, leachability and volatility of pesticides are directly related to organic matter content. The higher the soil organic matter content, the greater the soil’s ability to hold both water and adsorbed pesticides. In addition, sorbed pesticides are less accessible to microorganisms, which play an important role in the degradation of pesticides in soils [207,209].

Pesticides can decline populations of beneficial soil microorganisms, which are relevant for plants to transform atmospheric nitrogen into nitrates that are further used by plants [210]. Therefore, pesticides can exert harmful effect on plants, hindering nitrogen fixation and leading to reduced plant growth. Animals may be poisoned by pesticide residues when they enter sprayed fields or nearby areas shortly after been applied. Populations of birds and mammals that feed on earthworms can be reduced because pesticides can kill earthworms [211]. Alternatively, pesticides can indirectly affect animals by eliminating essential food sources, which forces them to relocate, change their diet or starve [212]. Populations of different bird species declined in the last decades from loss of plant and invertebrate species on which the birds feed, as well as a result of egg-shell thinning, as occurred with Dichlorodiphenyldichloroethylene (DDE) [213].

On the other hand, pesticide surface runoff and further accumulation in bodies of water has harmful effects on aquatic life. When pesticides reach sufficient levels, zooplankton population density may be reduced, which is the main source of food for young fish [214]. In addition, repeated exposure to sublethal doses of some pesticides can cause physiological and behavioral changes that reduce fish populations, such as abandonment of nests and broods, decreased immunity to disease and decreased predator avoidance. Furthermore, the application of herbicides to bodies of water can kill plants on which fish depend for their habitat. Further degradation of dead plants can kill fish as a result of consuming water’s oxygen [215].

### 5.4. Monitoring Programs for Pesticide Residues in Food: Risk Assessment

The use of pesticides on crops might pose a risk to consumers because of exposure to residues that remain in fruit or vegetable produces after crop treatments. National and international monitoring programs are in place to measure the occurrence of pesticide residues in foods. These programs are primarily focused on finding exceedances of established MRLs and also on providing representative data to assess health risks for consumers. Dietary risks for the population due to pesticide residues in foods are assessed based on food monitoring data, consumption surveys, and toxicological reference values for each pesticide.

Within the framework of the 2015 European Union Coordinated Program (EUCP) under Regulation (EC) No 400/2014, reporting EU countries analyzed 10,884 samples of 11 different food products. In particular, from 1045 samples of olive oil analyzed, 84.5% had no quantifiable pesticide residues, whereas 15.5% contained one or several pesticides in quantified concentrations. Multiple residues were found in 4.2% of olive oil samples. The overall quantification rate slightly decreased as compared to the year 2012, where 22% of olive oil samples contained pesticide residues. In total, 29 different pesticides were found in the olive oil samples analyzed in concentrations equal to or greater than the limit of quantification (LOQ). The active substances most frequently found were chlorpyrifos (7.3% of the tested samples), cypermethrin (5.3%), phosmet (4.5%) and dimethoate (1.7%). Compared to the 2012 EUCP, the same pesticides were found, but the quantification rates of chlorpyrifos and terbuthylazine decreased, particularly terbuthylazine (from 12% to 0.4% of the samples) [216].

Residue concentrations for olive oil require the application of a default processing factor of 5 to recalculate the legal limits set for unprocessed olives to olive oil under Regulation (EC) No 396/2005. This factor assumes that 5 kg of olives are used to produce 1 kg of oil and that a complete transfer of the residues to oil occurs. Although the residue concentrations exceeded the recalculated MRL only for 0.1% of olive oil samples analyzed under the 2015 EUCP, no exceedance of the acute reference dose (ARfD) was identified. Moreover, the long-term dietary exposure to the pesticides covered by the EUCP was unlikely to pose a health risk to consumers, as a wide safety margin to the toxicological reference value was observed [216].

The MRL exceedance rate for unprocessed food products under the 2015 EUCP and national programs showed that table olives presented the highest percentage of MRL exceedances (34.5%). In contrast, the results for processed products indicated that 4.8% of processed table olives exceeded the MRLs [217]. Olive crops devoted to producing table olives cover a far smaller area than those producing olive oil. For instance, less than 6% and 3% of the total olive grove area in Spain and Italy, respectively, are devoted to table olive production [217].

### 5.5. Sustainable Use of Pesticides

Around 50% of chemical pesticides are thought to be overused [218]. This overuse has led to the development of resistance, environmental pollution, toxicity to beneficial organisms and risks to human health. Since the 1990s, the United States and the EU implemented programs to update risk assessments for pesticides in use, which has led to the replacement of acutely toxic organophosphate and carbamate insecticides by newer pesticides with similar pesticidal efficiency and minimum side effects for non-target organisms [213].

EU policy sought to implement sustainable agricultural practices and to reduce reliance on pesticides by designing and implementing more integrated approaches towards the use of pesticides without affecting the competitiveness of the EU’s agriculture. The sustainable and safe use of PPP and the application of Integrated Pest Management (IPM) criteria should result in minimal use of chemical pesticides and strictly necessary employment (e.g., when other alternatives are ineffective). IPM relies on the principle of sustainable intensification, that is, the use of methods and practices to maintain or improve agricultural productivity without adverse environmental impact on insect, birds and mammals, and avoiding the cultivation of more land. IPM systems also entail an array of ecosystem goods and services beyond pest control, increasing general resilience at farm and landscape scales [197].

The Directive 2009/128/EC on the Sustainable Use of Pesticides aims to reduce the impact of pesticides on human health and the environment. To this end, EU Member States implemented National Action Plans to promote low-pesticide-input pest management and non-chemical alternatives to pesticides, including both IPM and organic farming. Indeed, the EU has experienced an upward trend in organic farming, where the total organic area increased from 5.6% of the total agricultural area in 2012 to 6.7% in 2016 [200]. This directive bound Member States to adopt measures to minimize off-site pollution from spray drift, drain flow and runoff in order to protect the aquatic environment and drinking water.

Another key feature of IPM is the use of biopesticides (also called biological pesticides). These compounds are inherently less toxic than conventional pesticides and generally affect only the target pest and closely related organisms, such that they are virtually non-toxic to people and the environment. Biopesticides consist of naturally occurring substances derived from animals, plants, bacteria, fungi and minerals used to control agricultural pests by means of specific biological effects. They include biochemical pesticides (i.e., azoxystrobin, azadirachtins, spinosads, and avermectins) or microorganisms (microbial pesticides, i.e., Bacillus thuringiensis), and pesticidal substances expressed in genetically modified crops (plant-incorporated protectants or PIPs). Biopesticides are effective in very small quantities and decompose quickly without leaving harmful residues. Therefore, biopesticides result in lower exposures and less environmental impact than conventional pesticides. Although biopesticides need to be approved and registered as such in most countries, fewer data are required for their registration as they pose fewer risks than conventional pesticides [201].

## 6. Other Health Properties of EVOO

This paper was not intended to be a detailed description of all the health effects attributed to the consumption of virgin olive oils. Indeed, we meant to report the outcomes of a conference attended by several worldwide experts. The conference discussed a limited number of pathologies. However, we would like to place on record that their extensive health effects have been highlighted in other recent articles [219,220,221,222,223], some of which make special reference to the role played by minor components [224,225,226]. This article follows the report of the II International Conference on Virgin Olive Oil and Health published in 2010 [227]. The third edition of the congress highlighted the major advances in the field, which we are reporting. Further important areas of investigation, such as memory and cognitive decline [228,229,230] will be the subject of future meetings.

## 7. Conclusions

The main conclusions of the meeting, as agreed upon by conveners were as follows:

1. The MedDiet is an excellent model of healthy eating. Olive oil, particularly extra-virgin olive oil, is the main and most characteristic component of the MedDiet. Indeed, without the use of olive oil, applying the label of “MedDiet” to another dietary pattern would represent a fairly inconsistent definition.
2. One of the greatest public health challenges worldwide is the obesity pandemic. There are sufficient studies reporting that the use of virgin olive oil as the only culinary fat, ingested in a moderate and continuous way, was associated with a reduced body mass index. Long-term randomized trials are warranted to confirm this observation.
3. Hypertension is the major risk factor for cardiovascular disease worldwide. The available randomized trials indicate that virgin olive oils reduce blood pressure and thus, the global cardiovascular burden of disease and its associated pharmaceutical costs.
4. Virgin olive oils have anti-atherosclerotic potential, favoring endothelial function and preserving blood pressure, maintaining lipoprotein functionality, exerting anti-inflammatory and antioxidant effects, and modulating gene expression in several tissues to maintain proper homeostasis.
5. Nevertheless, the type of olive oil should be considered when providing recommendations to the population because additional benefits can be conferred when the phenolic content of olive oil is high.
6. Epidemiological studies are concordant in supporting that a diet where virgin olive oils are the foremost source of fat is associated with chemoprevention. Animal studies are suggestive of a preventive effect of olive polyphenols and many in vitro studies are clarifying their mechanisms of action. However, the relevance of such data is often weakened by the use of non-physiological concentrations and doses. Human studies on chemoprevention with a single nutrient are nearly impossible to carry out. However, the advice to use olive oil as the principal source of visible fat to lower cancer risk rests on solid accumulated observations.
7. The Mediterranean diet—as an overall dietary pattern—has been shown to be associated with reduced risk of postmenopausal breast cancer in both observational human studies and a randomized trial. Moreover, observational studies suggest that specifically virgin olive oils may play a role in the prevention of postmenopausal breast cancer.
8. The Mediterranean diet has also been suggested to reduce the risk of colorectal cancer; nevertheless, the evidence is limited to observational data in humans and to date, there is no large body of evidence on the specific protective role of virgin olive oils on colorectal cancer prevention.
9. Although virgin olive oils have the potential to reduce the risk of some types of cancer (primary prevention), we have no strong clinical evidence to support that they can affect the long-term progression of pre-malignant or cancerous lesions after diagnosis (treatment).
10. Experimental studies have confirmed significant anti-inflammatory and immunomodulatory effects of dietary virgin olive oils and its bioactive components supplementation in preclinical models of autoimmune diseases, such as inflammatory bowel disease, rheumatoid arthritis, systemic lupus erythematosus and sclerosis. Thus, the consumption of virgin olive oil and its minor constituents may acquire a great importance in nutritional therapy, especially in immunocompromised patients, and could be an alternative approach for the prevention and management of different immune-inflammatory diseases. However, future clinical studies are necessary to mechanistically define the effective doses in humans and the dose-dependence of their effects.
11. Chemical pollution is one of the main determinants of morbidity and mortality in the world and represents an increasingly important threat for humans and the environment. However, only regulated chemicals (i.e., pesticides, food additives and veterinary medicinal products) have been subject to pre-market evaluation by means of rigorous toxicological testing in Western countries since the past two decades. In contrast, this is not the case for food contaminants and we still need to implement ways to reduce or prevent food contamination. While there is no current evidence that chemical pollution represents a health concern regarding olive oil, we need to shift towards a sustainable production of olive oil in order to reduce the potential chemical burden associated with its production.
12. The use of pesticides is necessary to combat pests and diseases in olive groves in order to increase olive oil production in terms of quantity and quality. However, an inappropriate use of pesticides may pose health risks to humans, non-target species, and the environment. Although theoretically, pesticide residues may remain in olive oil, the European Union Coordinated Programme for pesticide residues in food (for the year 2015) showed that only approximately 0.1% of the olive oil samples analyzed exceeded the MRL currently adopted. This indicates that the consumption of olive oil is unlikely to pose health concern to consumers. An integrated pest management approach is being implemented in the European Union to reduce the use of pesticides and foster a sustainable use of these substances without impairing crop production. This approach is essential and will optimize food production while minimizing risks to humans and the environment, thus contributing to achieving the integral concept of healthy food.
13. Sustainable food production is indispensable and unavoidable to feed the growing population. Better agronomic practices are urgently needed to guarantee nutritious food that is accessible to everyone and respectful of the environment. This also applies to olive oil.

## Figures and Tables

**Table 1:** Overview of extra virgin olive oil (EVOO) and molecular and cellular mechanisms participating in the origin and progression of cardiovascular diseases.

Experimental Models	Design	Effects	References
EVOO and atherosclerosis			
High risk subjects from PREDIMED	Mediterranean diet with EVOO compared to low-fat diet	Low Intima-media thickness (IMT)	[118,119]
Apolipoprotein E-deficient mice	EVOO	Decreased atherosclerosis	[120,121]
Rabbit	Hydroxytyrosol or squalene	Decreased gingival vascular damage	[122]
Human plaques	Oleacin incubation	Reduced secretion of metalloproteinases	[123]
EVOO and endothelial dysfunction			
Patients with prediabetes and diabetes participating in the CORDIOPREV clinical trial	Mediterranean diet with high EVOO compared to the same diet with low EVOO	Improved endothelial function	[57,124]
Healthy men and women	MedDiet rich in EVOO vs. regular diet	Lower systolic blood pressure and improved endothelial function	[125]
High risk subjects from PREDIMED	Mediterranean diet with EVOO compared to low-fat diet	Lower diastolic blood pressure	[59]
Hypertensive rats	Diet enriched in EVOO compared to chow diet	Decreased systolic blood pressure. Decreased oxide nitric (NO) and 8-isoprostane	[126]
ECV304 incubated with high glucose and fatty acids	Incubation with phenolic compounds	Increased endothelial NO synthase phosphorylation and NO levels. Decreased endothelin-1	[127]
Wistar rats	EVOO with or without phenolic compounds compared to control animals	Decreased vascular endothelial adhesion molecule-1 and E-selectin	[128]
EVOO and transcriptomics			
PBMC of healthy and metabolic syndrome subjects	Acute intake of high phenolic EVOO	Less deleterious inflammatory phenotype	[129]
EVOO and plasma lipids			
Healthy subjects	50 g daily of extra virgin coconut oil, EVOO or unsalted butter for 4 weeks	EVOO decreased total cholesterol/HDL-C ratio and non-HDL-C compared with butter. EVOO and coconut oil resulted in similar results for both parameters	[130]
Postmenopausal women	Butter or EVOO	EVOO decreased total/HDL-cholesterol and triglycerides/HDL-cholesterol	[131]
Healthy subjects (men and women)	54 g of corn oil and EVOO for 21 days	EVOO increased non-HDL cholesterol compared to corn oil. No differences on HDL-cholesterol but APOA1 increased more with EVOO compared with corn oil	[132]
Healthy subjects	Post-prandial 2-h lipid profile of subjects consuming Mediterranean-type meal with 10 g of EVOO or corn oil	EVOO produced less increase of LDL-C and ox-LDL compared with the corn oil	[133]
High risk subjects from PREDIMED	Mediterranean diet with EVOO compared to low-fat diet	EVOO increased LDL resistance against oxidation and decreased degree of oxidized LDL	[134]
High risk subjects from PREDIMED	Mediterranean diet with EVOO compared to low-fat diet	EVOO increased cholesterol efflux capacity, decreased cholesteryl ester transfer protein activity and increased HDL ability to esterify cholesterol, paraoxonase-1 arylesterase activity, and HDL vasodilatory capacity resulting in a more functional HDL	[72]
Healthy European male volunteers	Subjects received 25 mL/d EVOO of high phenolic content compared to the same a phenolic–poor EVOO for 3 weeks	Increased cholesterol efflux capacity	[70]
Apolipoprotein E-deficient mice	EVOO at 7 µl/mouse/day for 2 months stimulated	EVOO increased cholesterol efflux rate from mouse peritoneal macrophages	[121]
High risk subjects from PREDIMED	Mediterranean diet with EVOO compared to low-fat diet	Decreased plasma ceramide levels	[136]
High risk subjects from PREDIMED	Mediterranean diet with EVOO compared to low-fat diet	Changed 20 lipid species	[137]
*EVOO and adipose tissue metabolism*			
3T3-L1 adipocytes	Hydroxytyrosol	Increased mitochondrial biogenesis	[138]
Mouse adipose tissue	Hydroxytyrosol	Increased glutathione-driven antioxidant enzymatic machinery	[139]

HDL= High density lipoprotein; LDL, Low density lipoprotein.
